# Formulation Strategies for Fungal Biocontrol of Gastrointestinal Helminths in Domestic Animals and Plant-Parasitic Nematodes: A Review

**DOI:** 10.3390/pathogens15050488

**Published:** 2026-05-01

**Authors:** Júlia dos Santos Fonseca, Tábata Alves do Carmo, Bianca de Oliveira Botelho Vital, Thalita Suelen Avelar Monteiro, Huarlen Marcio Balbino, Huarrisson Azevedo Santos, Vagner Tebaldi de Queiroz, Fabio Ribeiro Braga, Jackson Victor de Araújo

**Affiliations:** 1 Department of Epidemiology and Public Health, Federal Rural University of Rio de Janeiro—UFRRJ, Seropédica 23890-000, RJ, Brazil; huarrisson@yahoo.com.br; 2 Department of Veterinary Medicine, Federal University of Viçosa—UFV, Viçosa 36570-900, MG, Brazil; tabata.carmo@ufv.br (T.A.d.C.); bianca.vital@ufv.br (B.d.O.B.V.); 3 Microbiota Brasil LTDA, Viçosa 36576-400, MG, Brazil; thalita.monteiro@microbiotabrasil.com.br (T.S.A.M.); huarlen.microbiota@gmail.com (H.M.B.); 4 Department of Chemistry and Physics, Federal University of Espírito Santo—UFES, Alto Universitário, Alegre 29500-000, ES, Brazil; vagner.queiroz@ufes.br; 5 Laboratory of Experimental Parasitology and Biological Control, Vila Velha University—UVV, Vila Velha 29102-920, ES, Brazil; fabio.braga@uvv.br; 6 Faculty of Veterinary Medicine, Fluminense Federal University—UFF, Niterói 24020-141, RJ, Brazil; jvictor@ufv.br

**Keywords:** biological control, *Duddingtonia flagrans*, nematophagous fungi, nematodes, helminths, *Pochonia chlamydosporia*

## Abstract

Although microbial biopesticides are expanding rapidly, transforming nematophagous fungi into consistent and shelf-stable products remains a challenge. A key limitation is that fungal propagules must remain viable throughout production, storage, and delivery to ensure their efficacy in the field. This review examines formulation strategies that improve the stability, deployment, and performance of fungal biocontrol agents against gastrointestinal helminths in domestic animals and plant-parasitic nematodes. In veterinary systems, predatory fungi such as *Duddingtonia flagrans* primarily target infective larvae after surviving gastrointestinal transit and germination in feces. In contrast, ovicidal fungi, including *Pochonia chlamydosporia*, *Purpureocillium lilacinum*, *Trichoderma* spp., and *Mucor* spp., primarily act against helminth eggs and coccidian oocysts. This functional complementarity highlights the potential of combined fungal formulations to improve their control efficacy. We also discuss the currently available *D. flagrans*-based commercial products, BioWorma^®^ and Bioverm^®^, and the practical challenges associated with dosing, administration, and farm adoption. In agriculture, we show that the Brazilian market is dominated by solid fungal nematicides designed to reduce water activity and prolong shelf life, although liquid- and oil-based systems remain relevant for specific applications. Across both sectors, the review identified formulation design, rather than fungal species alone, as a critical determinant of product performance. Emerging advances, such as microencapsulation, UV-protective matrices, improved seed-coating biopolymers, nanobiotechnology, and fungal-derived bioactive products, indicate that future progress will depend on target-oriented formulations capable of increasing stability, controlled release, and resilience under environmentally variable conditions, including those imposed by climate change.

## 1. Introduction

The use of microorganisms to manage pests and diseases in agriculture has long been recognized, although biological products have become widely available only in the recent decades [1,2]. This expansion is largely due to advances in formulation technologies, which are essential for maintaining living microorganisms in a stable state with sufficient shelf life so that the biological product remains viable at the time of application and persists in the environment until it reaches its target [3,4]. Among these biological agents, nematophagous fungi have attracted attention because of their direct and indirect effects on various nematode species in both agricultural and veterinary systems [5,6]. These microorganisms act through mechanisms that include trapping infective larvae and parasitizing eggs, thereby helping to reduce environmental contamination and parasitic pressure [7,8,9,10].

**Figure 1:**
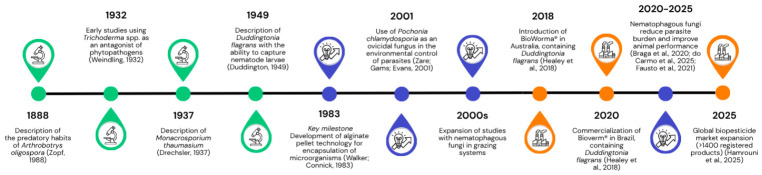
Timeline illustrating the development of fungal bioformulations for nematode control in agricultural and veterinary systems, highlighting key biological discoveries (green), advances in formulation technologies (blue), and the transition to industrial-scale production and commercial application (orange). Source: Weindling, 1932 [28]; Duddington, 1949 [19]; Zare, Gams, Evans, 2001 [29]; Healey et al. 2018 [13]; Braga et al. 2020 [30]; do Carmo et al. 2025 [31]; Fausto et al. 2021 [32]; Zopf, 1988 [20]; Drechsler, 1937 [21]; Walker and Connick, 1983 [11]; Hamrouni et al. 2025 [12].

The evolution of fungal bioformulations, from early biological discoveries to advances in formulation technologies and their translation into industrial applications, is illustrated in Figure 1. A major milestone for microbial bioformulations in agriculture and veterinary medicine was reported by Walker and Connick (1983) [11], who encapsulated fungal spores in sodium alginate pellets to improve their viability and environmental release. Subsequently, during the 1990s, biopesticides underwent regulatory and commercial expansion, with the EPA registering more than 100 microbial active ingredients. The global market currently offers more than 1400 biopesticide products [12], including commercial formulations such as BioWorma^®^ and Bioverm^®^, both of which are based on *Duddingtonia flagrans*. Under field conditions, these products have reduced the dependence on chemical anthelmintics, helped mitigate resistance, and promoted weight gain in livestock [13,14].

Initially developed for the management of plant pests and diseases, these technologies have since been adapted and refined for the biological control of parasites in animal production systems [15] (Figure 1). In agriculture, the fungi most commonly studied for the control of plant-parasitic nematodes include the predatory genera *Arthrobotrys* spp. and *Monacrosporium thaumasium* [16], as well as the ovicidal species *Purpureocillium lilacinum* and *Pochonia chlamydosporia* [7]. Recently, *Duddingtonia flagrans* has been investigated for this purpose [17]. Ovicidal fungi are the most common in commercial formulations because they are easier to mass-produce and are effective in controlling plant-parasitic nematodes under field conditions. Many of these fungi also promote plant growth [18].

In veterinary medicine, fungal bioformulations have emerged as an alternative for controlling gastrointestinal nematodes in production animals because of increasing anthelmintic resistance, concerns about drug residues, and environmental impact of synthetic chemicals. Promising agents include nematophagous fungi such as *Duddingtonia flagrans* [19], *Arthrobotrys oligospora* [20], and *Monacrosporium thaumasium* [21], which capture and destroy infective larval stages, as well as *Pochonia chlamydosporia*, which acts on eggs and females [7,8] (Figure 1). By acting in the environment, these fungi reduce pasture contamination and parasitic pressure on animals, thereby contributing to more sustainable control strategies [9,10].

Despite the growing number of studies and commercial products, there is still a lack of consolidated understanding of how formulation strategies influence the stability, viability, scalability, delivery, and efficacy of the active microorganism in fungal bioformulations under field conditions [3,22]. The performance of microorganism-based products is influenced by soil microbiology, insect pathology, and microbial interactions and may be adversely affected by environmental variability, reduced microbial survival, difficulties in large-scale production, and limited shelf life [23]. The use of solid or liquid systems [24], encapsulation technologies [25], and delivery approaches [26] are key formulation strategies that critically influence the large-scale implementation and long-term efficacy of biocontrol agents. Therefore, although numerous fungal species have demonstrated significant potential for nematode control [6], only a limited number have been successfully translated into commercially viable products [23,27].

This article reviews the development, formulation strategies, and applications of fungal bioformulations for controlling helminths in animals and phytonematodes in agriculture. It highlights the role of nematophagous fungi as sustainable alternatives to chemical control, as well as advances in formulation technologies that improve stability, viability, and field efficacy. This article also addresses the challenges related to production, shelf life, and commercialization, and discusses future perspectives for expanding the use of these biocontrol strategies.

## 2. The Use of Fungal Bioformulations in the Control of Helminths

### 2.1. Helminths in Domestic Animals—In the Veterinary Area

Fungal structures such as conidia and chlamydospores can pass intact through the gastrointestinal tract, concentrate in the feces, and germinate there, forming three-dimensional adhesive hyphal networks that capture infective L3 larvae through traps such as loops or nets [33]. This infection process involves intercellular communication mediated by bZIP transcription factors, hyphal fusion, and signaling through G-proteins and MAPK kinases, culminating in the penetration of the nematode cuticle by extracellular enzymes such as serine proteases and chitinases. These enzymes degrade chitin and cuticular proteins, enabling the internal digestion of the host [34,35]. Consequently, the parasite load in the environment is reduced, which in turn decreases the infection pressure on animals [32,36,37,38]. These microorganisms have demonstrated the ability to reduce helminth populations without causing adverse effects in host animals [37,39].

**Figure 2:**
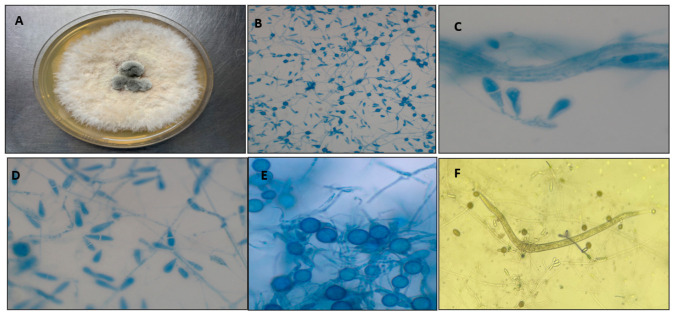
Morphological characterization and predatory activity of *Duddingtonia flagrans*. (**A**) Macroscopic appearance of the fungal colony on agar medium, showing a white, cottony aerial mycelium. (**B**,**D**) Photomicrographs displaying hyaline, ellipsoidal conidia stained with lactophenol cotton blue stain. (**C**) Details of conidial morphology (100× magnification). (**E**) Detailed view of chlamydospores, highlighting the thick-walled resistance structures characteristic of this species (100× magnification). (**F**) Biological interaction between the fungus and nematode, demonstrating the capture and predation of a *Panagrellus* spp. larva through mycelial traps (10× magnification)—Photograph by Prof. Fabio R. Braga and M.Sc. Carolina Magri Ferraz.

*Duddingtonia flagrans* is one of the main biological control agents of gastrointestinal parasitic nematode larvae. Over the past few decades, the high production of chlamydospores has been extensively studied (Figure 2), underscoring its commercial potential [40]. In addition, its predatory activity against infective larvae (L3) has positioned *D. flagrans* as one of the most promising biological control agents for pasture-based systems.

*Pochonia chlamydosporia*, *Mucor circinelloides*, *Purpureocillium lilacinum*, and *Trichoderma* spp. exhibit ovicidal activity against helminth eggs and coccidia oocysts. These fungi can adhere to, penetrate, and destroy the eggs [41,42]. Research also suggests that *P. chlamydosporia* may be effective against cestode eggs [43,44] and trematodes [45] in the environment. This broad-spectrum activity reinforces the relevance of ovicidal fungi as complementary tools to larvicidal species in integrated parasite management programs.

The success of fungal bioformulations depends on factors such as the route of administration, the intake of effective doses, and storage conditions that preserve fungal viability and biological activity [46]. Reported dosages vary among studies: *D. flagrans* is commonly administered at 10^6^ chlamydospores/kg body weight/day (Bioverm^®^: 1 g/10–100 kg) or 3 × 10^6^/kg (BioWorma^®^), with regimens ranging from twice weekly to daily for three to six months. Low-dose regimens (<10^5^/kg) showed reduced efficacy without toxicity, whereas standardized dosages of 10^6^/kg administered twice weekly optimized larval reduction (>80%) in pastures [13,39]. Although several administration regimens have produced good results, long-term protocols involving at least one oral administration twice weekly are expected to provide the best parasite control [39]. Moreover, standardizing the dosage and delivery protocols is essential to ensure reproducibility across studies and consistent field performance.

The combination of fungi with different mechanisms of action may generate complementary or even synergistic effects in biological control [47,48,49]. Therefore, the association between species with ovicidal and larvicidal activities is particularly promising. These combinations have also shown resistance to the nutritional pellet manufacturing process, enabling effective control of helminths in domestic animals in pastures and captive wild animals [42,50]. For example, a pelleted combination of *Arthrobotrys cladodes* and *P. chlamydosporia* showed greater nematicidal activity in cattle than either fungus alone (92% versus 78% larval reduction), suggesting a synergy between larvicidal and ovicidal mechanisms. However, this effect has not been consistent across studies; one study reported only additive effects in sheep [51], and *Arthrobotrys* viability may decline by 30–50% during joint pelleting because of nutritional competition [52].

Commercial products based on nematophagous fungi have been developed as innovative biological control strategies against nematodes. Bioverm^®^, for example, is a powder containing 10^5^ chlamydospores of *Duddingtonia flagrans* per gram. The biological control potential of *D. flagrans* has been widely demonstrated, with larval reductions above 90% for *Strongyloides papillosus* and *Haemonchus contortus* in sheep [30]. Another example is BioWorma^®^, a commercial product manufactured in Australia and formulated with *D. flagrans* chlamydospores. It is administered at a dose of 3 × 10^4^ chlamydospores/kg body weight. Even at lower doses than those commonly used in experimental studies, BioWorma^®^ significantly reduced the number of viable nematode larvae in horses, goats, and cattle [13]. These findings support the feasibility of implementing fungal-based products in diverse production systems and host species.

**Figure 3:**
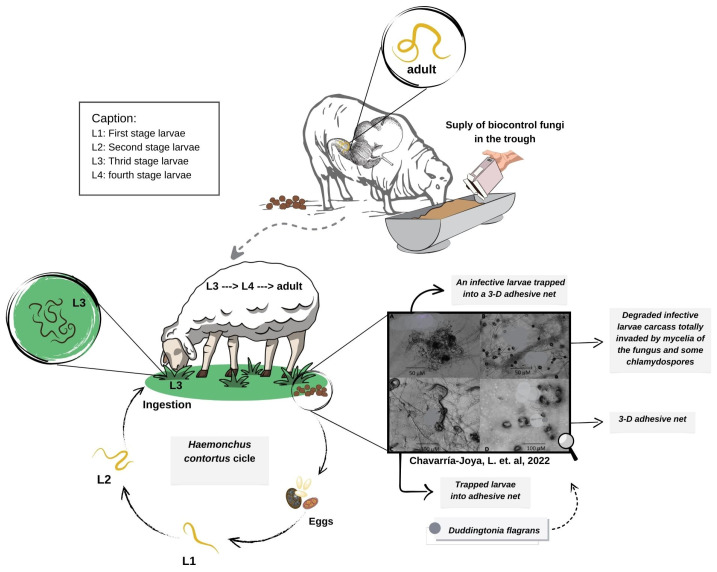
Dynamics of helminth biocontrol in production animals using helminthophagous fungi (from author’s projects). Source: Faria et al. 2025 [53]; Chavarría-Joya et al. 2022 [54].

Administering fungal bioformulations through dietary supplementation or commercial formulations ensures the survival of chlamydospores as they pass through the digestive tract of animals (Figure 3). This represents a significant advance in the sustainable control of helminthiasis [31]. The development of fungal formulations for biological control is a key step towards commercializing these microorganisms. However, economically viable production of fungal material is essential, including optimization of fermentation conditions, formulation stability, and shelf life, as well as being an important step towards enabling the commercial production of helminthophagous fungi [52].

### 2.2. Phytonematodes—Agricultural Area

Plant-parasitic nematodes pose significant phytosanitary challenges to global agriculture. They cause significant productivity losses in a wide range of crops. These phytopathogens are often overlooked because of their microscopic nature and the fact that they are hidden in the root system of plants. They are also difficult to control because they dwell in the soil. Biological control stands out among the practices used in integrated nematode management.

The potential of microorganisms for nematode control in agriculture has been recognized for several years. However, significant advances in formulations have led to a substantial increase in access to biological products in the past few decades. Improvements in this field ensure that the active ingredient, a living microorganism, maintains stability and an adequate shelf life to guarantee its viability at the time of application. It also preserves the ability of microorganisms to act in the environment until they reach the target organisms.

**Figure 4:**
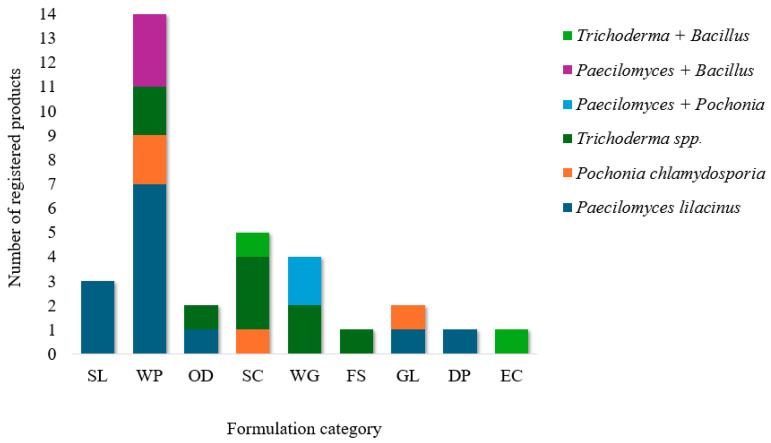
Distribution of registered biological control agents across different commercial formulation types. The columns represent the number of registered products for each formulation: Soluble Concentrate (SL), Wettable Powder (WP), Oil Dispersion (OD), Suspension Concentrate (SC), Water-Dispersible Granules (WG), Flowable Concentrate for Seed Treatment (FS), Emulsifiable Gel (GL), Dry Powder (DP), and Emulsifiable Concentrate (EC).

Most nematophagous fungi can be mass-produced on solid culture media. Because fungal propagules must be kept stable without germination or hyphal branching, which requires careful control of water activity, solid formulations are more common when fungi are used as the active ingredient (Figure 4) [3,22].

In fungi, spore germination and hyphal branching readily occur in the presence of water. Consequently, it is difficult to halt microbial development in liquid formulations, unlike in endospore-forming bacteria [23]. Therefore, some companies have developed formulations based on blastospores, which are produced by hyphal budding and can often be generated in higher quantities than spores obtained on solid media [55].

In recent years, highly concentrated liquid media have been used, although these formulations generally have a shorter shelf life. Bioformulations for the control of plant-parasitic nematodes have evolved from simple powders and suspensions to systems that protect microorganisms from environmental stress and accelerate their establishment after their application. These systems include adjuvants, such as UV protectants, antioxidants, and nutritional additives, which improve persistence and field performance [56]. Microencapsulation using polysaccharide matrices (e.g., alginate/chitosan, sometimes combined with nanocellulose) has been explored to increase viability, controlled release, and shelf life [25]. In addition, microcapsules containing UV absorbers have shown greater persistence under solar radiation and improved thermal stability in biopesticides, establishing useful technological standards for the development of microbial formulations [57]. For plant-parasitic nematodes, seed-coating formulations with *Pochonia chlamydosporia* have shown that biopolymers, such as gellan and xanthan gum, enhance blastospore viability after drying and that refrigeration extends storage stability [58]. However, although blastospores in liquid formulations facilitate high loading and seed coating, they are more sensitive to humidity and temperature than those produced on solid media. Therefore, controlling water activity, using active packaging, and maintaining low temperatures are key to extending the shelf life of these products [24].

Because plant-parasitic nematodes are soil-dwelling pathogens, application strategies that place biocontrol agents close to the root zone are essential for effective management. Two important strategies are the application in planting furrows and seed treatment, the latter being especially preferred by grain growers because of its operational practicality and ability to protect seedlings immediately after germination [5,19,59]. To make this practice feasible, companies have invested in formulations that improve the adhesion and stability of microorganisms in seeds.

The availability of these products and their use in agriculture have increased in countries across continents. Brazil is a major player in this scenario. Therefore, we present data on fungal-based microbiological nematicides that are registered in Brazil.

#### 2.2.1. Fungus-Based Formulations for Controlling Plant-Parasitic Nematodes

**Figure 5:**
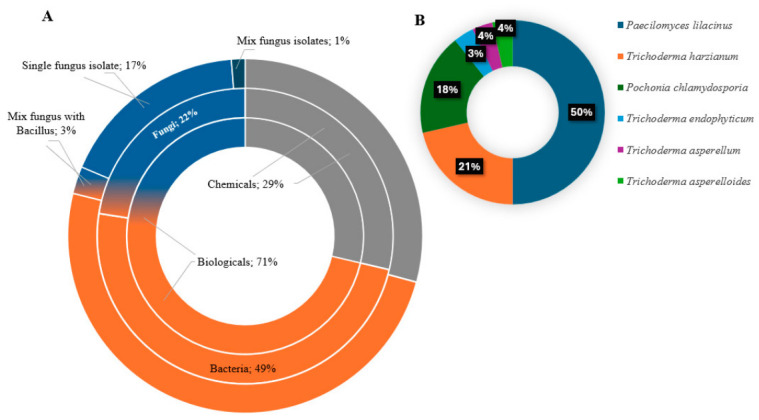
Distribution of chemical and biological nematicides registered in Brazil based on data from the Agrofit database. (**A**) Proportion of registered products categorized as chemical or biological (inner ring). Biological products are further subdivided into bacterial- and fungal-based products (middle ring). Fungal-based products are classified according to formulation type (outer ring) as single isolates, mixtures of fungal isolates, and combinations involving *Bacillus*. (**B**) Relative distribution of fungal species used in the registered biological nematicides. Data were obtained from the Agrofit system (Ministério da Agricultura e Pecuária, 2025) [60].

In Brazil, 71% of registered nematicide products contain microorganisms as active ingredients. Bacteria accounted for the largest proportion of these products (68%) (Figure 5A), mainly because of the ease and rapid turnaround of high-volume manufacturing. Fungi represented the remaining 32% and may occur as isolates or in combination with bacteria. The fungi most commonly used are *Paecilomyces lilacinus*, *Pochonia chlamydosporia*, and species of the genus *Trichoderma* (Figure 5B). Given the importance of these microorganisms in the nematicide market, their processing and availability to farmers are critical issues.

Product formulation is the process of converting an active ingredient into an optimized physical form that enhances its biological performance while enabling efficient storage and application [61]. This approach is essential for the widespread adoption of biological control agents, ensuring that the correct dosage is administered at the right place and time. Typically, the precise formulation composition is a trade secret that ensures product exclusivity and performance. Although the global market has historically led in advanced stabilization techniques, Brazil has rapidly emerged as a powerhouse in this sector, adapting formulations to the particularities of tropical climates. Unlike the standardized global approach, Brazilian bioproducts often prioritize resistance to high ultraviolet radiation and thermal fluctuations, leveraging the country’s enormous production scale to achieve cost-competitiveness.

A biological control product consists of three main components: the first is the active ingredient, which is a microorganism responsible for the biological action. This microorganism is typically represented by conidia, chlamydospores, blastospores, and microsclerotia [62]. The second component is the vehicle or carrier, which is an inert substance that provides physical support and assists in transporting the active ingredient to the target site. Solid carriers, such as talc, starch, wheat flour, and sterilized grains, are commonly used in *Trichoderma*, *P. chlamydosporia*, and *P. lilacinus* formulations because they reduce water activity and prolong the viability of the formulations during storage [26,63]. Liquid vehicles, such as concentrated suspensions or emulsions, offer advantages in application but require stabilizers to maintain homogeneity and prevent loss of cell viability [63]. The third component consists of adjuvants that increase the stability, efficacy, and practicality of the products. This group includes additives such as antioxidants, cryoprotectants, and osmoprotectants, including glycerol and trehalose. These additives preserve the membrane and proteins during drying and storage. Studies on *T. harzianum* have shown that adding glycerol to solid formulations can prolong viability for up to 12 months [64]. Supplementation with protective sugars also had a positive effect on desiccation tolerance in *Metarhizium* and *Beauveria spores* [65].

Surfactants are classified as adjuvants. They modify the surface properties of the spray mixture and increase contact between microorganisms and hosts or substrates. Spreaders improve spray coverage on leaves and roots, and wetting agents delay the drying of the deposit, which prolongs the wetting time and promotes conidial germination [66]. Dispersants reduce agglomeration and promote the uniform distribution of particles, which is particularly useful in liquid formulations [67]. Additionally, photoprotective adjuvants, such as lignin and plant extracts, have been evaluated for their ability to protect *Pochonia* and *Trichoderma* spp. spores from UV radiation. These adjuvants have shown promise in increasing the efficacy of formulations under field conditions [68]. These components, which are added in small quantities to improve the formulation, can influence the stability and performance of the product.

Biological product formulations can be found in either liquid or solid form. Although liquid formulations are generally simpler and more economical to produce, solid formulations are more stable during storage. In this context, the determining factor is water activity (a_w), which regulates microbial metabolism and the rate of cell deterioration. Reducing a_w limits metabolic and enzymatic reactions, favoring the dormant state of propagules and prolonging product viability [24,62]. Controlled drying or freeze-drying is widely used to achieve a_w levels below 0.3, which corresponds to a moisture content of less than 5%. This condition is considered ideal for product stabilization [68]. However, susceptibility to propagules varies by type. For example, blastospores are more sensitive to water stress and require the addition of osmoprotectants, such as glycerol and trehalose, during production or formulation to prevent irreversible damage to cell membranes [63,64].

Another critical point is the choice of carrier, as it can directly impact the maintenance of a_w and the physical protection of the microorganisms. Carriers with low hygroscopicity and high adsorption capacities contribute to the stability of the formulations [26]. Additionally, active packaging containing desiccants or films with low water vapor permeability reduces moisture absorption from the environment, thereby slowing the loss of viability [69]. Storage at low temperatures (approximately 4 °C) is also recommended because lowering the temperature slows degradation reactions, significantly extending the shelf life of fungal propagules, such as conidia and chlamydospores [70,71].

Several types of formulations are used in Brazil for nematode control in fungus-based products. The types are:

- Liquid: oil dispersion (OD), soluble concentrate (SL), and concentrated suspension (SC).
- Solid: wettable powder (WP), dispersible granules (WG), emulsifiable concentrate (EC), concentrated suspension for seed treatment (FS), and powder for preparation of paste in water (WS).
- Other: emulsifiable gel (EG).

#### 2.2.2. Liquid Formulations

The efficacy of liquid biocontrol agents is intrinsically linked to their chemical and biological composition, which typically consists of cell suspensions enriched with specific additives. The incorporation of stabilizers, surfactants, dyes, and nutrients is fundamental to prolonging the viability of bioproducts, as well as optimizing the adhesion, dispersion, and photoprotection properties of microbial cells [72]. Liquid formulations are primarily categorized into oil dispersions (OD) and aqueous formulations (SL and SC), each exhibiting distinct performance profiles. Although aqueous variants offer lower production costs and ease of handling, they demonstrate operational limitations in the field, such as high evaporation rates and vulnerability to ultraviolet radiation degradation. Conversely, OD formulations provide enhanced leaf adhesion and rainfastness, thereby increasing agent persistence under environmental stress. However, the higher viscosity of these formulations can induce spray nozzle clogging, necessitating rigorous stability protocols to mitigate phase separation during storage [73,74,75].

Oil-based formulations are prepared by combining processed microbial cultures with oils (mineral or vegetable) and adding emulsifiers and surfactants to facilitate their subsequent dispersion in water. The choice of oil was carefully considered to ensure that it was non-toxic to microorganisms, crops, humans, and animals. Oil-based formulations are widely accepted as particularly suitable for foliar spraying under low-humidity conditions because the oil phase provides a protective layer that extends microbial viability [76]. However, the environmental conditions at the time of application must be monitored to mitigate phytotoxicity.

In contrast, aqueous formulations involve separating the biomass and suspending it in a water-based solution that acts as a carrier [77]. Adjuvants can be added to this solution to ensure the physical stability and integrity of the formulation during its application.

Although liquid formulations bypass the need for costly drying infrastructure, they impose important logistical constraints. Managing large volumes, together with the risk of microbial contamination, often requires strict cold storage and transport. These requirements increase operational costs and result in a shorter commercial shelf life and lower inventory flexibility, creating a substantial barrier to large-scale market adoption.

#### 2.2.3. Solid Formulations

Solid formulations can simplify logistics because the suspension is prepared only at the point of use, thereby reducing transportation constraints during production and distribution. However, this advantage shifts the technical challenge to the stabilization stage, where careful control of separation and drying, using methods such as spray drying or freeze-drying, is essential to avoid compromising microbial viability.

Wettable powders (WPs) remain widely used in nematode biocontrol, particularly in fungus-based formulations, because they are relatively simple to manufacture and cost-effective. Their production is facilitated by the fact that fungal sporulation is favored in solid-state fermentation on cereal-based substrates; subsequent dehydration and grinding help ensure stability until reconstitution at the point of use. However, although WPs facilitate large-scale processing, the mechanical and thermal stress associated with processing often requires additional protection for microorganisms and may reduce environmental persistence. This conventional approach contrasts with more advanced technologies, such as encapsulation. While WPs offer immediate bioavailability at a lower cost, encapsulation provides a protective microenvironment that shields biocontrol agents from UV radiation and desiccation. Although encapsulation requires greater production complexity and capital investment, it enables the synchronized and controlled release of bioproducts, representing a significant advance over the rapid and often short-lived action of wettable powders.

The combination of active ingredients, carriers, and adjuvants must ensure the stability and viability of microorganisms during storage, as well as ease of application in the field. Each type of formulation has its own advantages and challenges; however, advances in drying techniques, packaging, and additives have increased the potential for adopting these products. In this context, developing more efficient formulations adapted to Brazilian agricultural conditions is essential for establishing biological control as a practical and competitive tool for managing phytonematodes.

## 3. The Veterinary Field’s Commercial Products, Their Economic Viability, and Future Prospects

Unlike the agricultural sector, which offers several commercial products, the veterinary market currently has only two commercial formulations containing *Duddingtonia flagrans*. These are Bioverm^®^ (AC001, Cinergis/GhenVet Saúde Animal, Paulínia, Brazil), marketed in Brazil, and BioWorma^®^ (NCIMB 30336, BioWorma, Sydney, Australia), marketed in Australia, New Zealand, and the United States. Both products contain chlamydospores of this fungus and are administered as feed to production animals [31]. Given the broad activity of nematophagous fungi and related organisms [78], a wider market availability of these products would be desirable.

Regarding economic viability, Bioverm^®^ is recommended for daily use and costs approximately US$12 per kilogram. It contains 10^6^ chlamydospores per gram and is formulated on a rice-bran base. The recommended dosage is 1 g for every 10 kg of live weight in small ruminants and 1 g for every 100 kg of live weight in large ruminants and horses. Most of its efficacy against the free-living stages of nematodes (e.g., eggs and larvae in the environment) occurs within the first three months of its application [79,80]. For an adult bovine weighing 600 kg, treatment over three months would cost less than USD 7.20, suggesting that the product may be economically feasible. De Oliveira et al. [79] reported a weight gain of 54.3 kg in the treated group compared with the control group after six months in growing beef cattle. The study was conducted under grazing conditions with Bos taurus indicus; mineral supplementation was provided only to growing animals, including males aged six–ten months, with an average initial weight of 180 kg. Although this represents one of the most basic livestock production systems worldwide, the observed weight gain was significant. In addition to improving growth, the use of nematophagous fungi may reduce the need for anthelmintic treatments, decrease gastrointestinal damage associated with infection and ingestion of infective larvae, and minimize environmental and carcass contamination linked to the use of chemical treatments.

**Figure 6:**
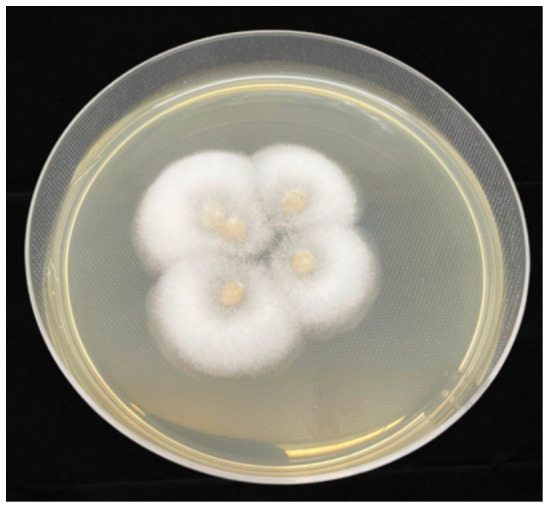
Sodium alginate pellets containing *Duddingtonia flagrans*, showing the formation and distribution of the fungus within the solid formulation (photograph by Bianca de Oliveira Botelho Vital, Vagner Tebaldi de Queiroz, and Jackson Victor de Araújo).

Several nematophagous fungi, including *Monacrosporium thaumasium*, *Arthrobotrys robusta*, and *Duddingtonia flagrans* (Figure 6), *Mucor circinelloides*, *Pochonia chlamydosporia*, and *Paecilomyces lilacinus* have been formulated into sodium alginate pellets, feed cakes, feed bars, gelatin matrices, and cereal grains [81,82]. These fungi exhibit broad, non-selective activity against eggs and larvae in the environment [78].

Many studies have produced these fungi on solid substrates, such as cereal grains, which are subsequently fed to livestock. In the 1990s, a Danish research group, in collaboration with Christian Hansen A/S, launched a commercial product containing *D. flagrans* in barley. This product was distributed throughout Europe but is currently unavailable in the market. Intraruminal cakes were also considered but abandoned because of the lack of a viable method for slow-release delivery. Sodium alginate pellets have been successfully applied in several domestic animal studies, offering a stable solid formulation that passes slowly through the gastrointestinal tract; however, these formulations are not yet commercially available.

Palatable feed bars containing 2 × 10^6^ spores of *Mucor circinelloides* and an equal dose of *D. flagrans* per kilogram have been administered to horses, maintaining strongylid egg counts below 200 eggs per gram of feces for 16 months [39], suggesting a practical approach for animals with selective feeding behavior. Similarly, formulations designed for dogs have been explored; chlamydospores of *M. circinelloides* and *D. flagrans* were incorporated into edible jellies and administered three times per week for 17 months to 18 dogs [83]. Each milliliter of jelly, prepared with chicken broth prior to cooling, contained 5–7.5 × 10^3^ chlamydospores of each fungus. Furthermore, plant- and chitin-rich residues, such as shrimp shells and mushrooms, have been successfully used to cultivate *P. chlamydosporia*, a nematophagous fungus effective against *Meloidogyne* spp. In submerged fermentation, this species can metabolize chitosan and produce ethanol and bioactive metabolites, in addition to its nematicidal activity, thereby expanding its technological applications [84].

The application of nanotechnology in biological control represents a promising and emerging approach. Nanoparticles (NPs), particularly those biosynthesized by fungi, have demonstrated antiparasitic activity against helminths and their environmental stages. For instance, NPs derived from *Duddingtonia flagrans* have shown effects on infective larvae (L3) of equine cyathostomins, bovine strongylids, and eggs of parasites, such as *Ancylostoma caninum* and *Toxocara canis*, under laboratory conditions [85,86,87,88]. Other nematophagous fungi, such as *Monacrosporium thaumasium*, have also exhibited activity against intermediate hosts, including *Biomphalaria glabrata*, in controlled laboratory experiments [89]. Despite these promising findings, most evidence is limited to in vitro assays or experimental systems, with restricted validation under field conditions. The mechanisms underlying the antiparasitic activity of fungal-derived NPs remain incompletely understood, particularly regarding their interactions with complex environmental matrices and their potential effects on non-target organisms. Additional limitations include environmental instability (sensitivity to UV radiation, temperature fluctuations, and interactions with organic matter), which may affect the efficacy under real-world conditions. Although some studies suggest that biosynthesized NPs may be biodegradable and environmentally compatible, comprehensive ecotoxicological assessments are scarce [90,91]. Furthermore, challenges associated with large-scale production, standardization, and economic feasibility must be considered before practical implementation. While fungal bioproducts may incur higher initial costs and require more technical expertise than conventional nematicides or anthelmintics, their potential benefits, including reduced environmental persistence, lower resistance risk, and higher ecological compatibility, may offset the investment over time. Regulatory frameworks for nanoparticle-based biocontrol agents are still under development, which may delay their adoption in agricultural and veterinary systems. Therefore, although nanobiotechnology represents a potentially valuable complementary strategy, further studies are required to validate its safety, efficacy, and economic feasibility under field conditions.

The relative ease of fungal cultivation supports the technical feasibility of producing bioactive compounds, including extracellular enzymes (e.g., proteases and chitinases), secondary metabolites, and metal-based NPs. However, scalability, batch-to-batch consistency, and stability under field conditions remain significant challenges. Fungal-derived compounds have attracted considerable attention for controlling parasitic helminths in domestic animals and phytonematodes in agriculture because of their chemical diversity and multifunctional modes of action. Secondary metabolites, including alkaloids, terpenoids, polyketides, and non-ribosomal peptides, exhibit antimicrobial, antifungal, and antiparasitic activities. Although these compounds are promising alternatives to synthetic anthelmintics and nematicides, their efficacy, selectivity, and environmental persistence require validation under field conditions [92]. Metabolites of *Pochonia chlamydosporia* with structural similarity to ketamine have demonstrated antiparasitic activity comparable to albendazole in experimental systems; however, this comparison should be interpreted with caution because of differences in experimental design, pharmacodynamics, and safety profiles [93].

Extracellular enzymes play a central role in host–parasite interactions by degrading the eggs and larval cuticles. Proteases, chitinases, and associated enzyme complexes have demonstrated ovicidal and larvicidal activities in vitro, with reported efficiencies ranging from 20% to 100%, depending on the target species and experimental conditions. Nonetheless, enzyme stability, delivery systems, and persistence in complex environmental matrices remain limiting factors for practical applications. These enzymes may be used as standalone agents or incorporated into more complex bioformulations, although their performance under field conditions remains insufficiently characterized [91].

Evaluating virulence-associated traits, such as thermotolerance, is essential for the safe development of fungal-based biocontrol strategies. Thermotolerance influences both efficacy and biosafety, particularly under climate change scenarios that could favor the emergence of opportunistic and pathogenic strains [94,95]. Therefore, a detailed understanding of these physiological traits is crucial to ensure that fungal bioformulations are effective against target parasites and safe for non-target organisms, including humans, animals, and plants [27].

## 4. Conclusions

Fungal bioformulations are promising and sustainable options for controlling gastrointestinal helminths in animals and plant-parasitic nematodes. Their performance is closely linked to formulation design, particularly the ability to maintain propagule viability and ensure effective delivery under field conditions. Solid formulations, especially those based on chlamydospores, continue to offer greater stability, whereas approaches such as microencapsulation provide additional protection and more controlled release. The combined use of fungi with different modes of action also appears to improve the overall efficacy.

However, several limitations remain. There is still no clear standardization of dosing or application protocols, and long-term field studies are relatively rare. In addition, the interactions between fungal agents and environmental factors are not yet fully understood. Questions related to biosafety, environmental impact, and economic feasibility, particularly for newer technologies such as nanoparticle-based systems, require further investigation.

Future studies should focus on improving formulation consistency and adapting products to different production systems. More field-based studies across diverse environments are needed to confirm the performance under practical conditions. Advances in formulation technologies should be accompanied by careful evaluation of safety and environmental impact. Integrating fungal agents into broader parasite control strategies, along with selecting strains that perform well under variable conditions, will be important steps.

Overall, progress in this field will depend on balancing formulation quality, biological effectiveness, and practical applicability, thereby supporting the wider use of fungal bioformulations in sustainable parasite control.

## Data Availability

Data sharing is not applicable to this article because no datasets were generated or analyzed in this study.
